# Granulocyte-Colony Stimulating Factor (G-CSF) for stroke: an individual patient data meta-analysis

**DOI:** 10.1038/srep36567

**Published:** 2016-11-15

**Authors:** Timothy J. England, Nikola Sprigg, Andrey M. Alasheev, Andrey A. Belkin, Amit Kumar, Kameshwar Prasad, Philip M. Bath

**Affiliations:** 1 Vascular Medicine, Division of Medical Sciences and GEM, School of Medicine, University of Nottingham, UK; 2 Stroke Trials Unit, Division of Clinical Neuroscience, School of Medicine, University of Nottingham, UK; 3 Institute of Medical Cell Technologies, Yekaterinburg, Russia; 4 Department of Neurology, Neurosciences Centre, All India Institute of Medical Sciences, New Delhi, India

## Abstract

Granulocyte colony stimulating factor (G-CSF) may enhance recovery from stroke through neuroprotective mechanisms if administered early, or neurorepair if given later. Several small trials suggest administration is safe but effects on efficacy are unclear. We searched for randomised controlled trials (RCT) assessing G-CSF in patients with hyperacute, acute, subacute or chronic stroke, and asked Investigators to share individual patient data on baseline characteristics, stroke severity and type, end-of-trial modified Rankin Scale (mRS), Barthel Index, haematological parameters, serious adverse events and death. Multiple variable analyses were adjusted for age, sex, baseline severity and time-to-treatment. Individual patient data were obtained for 6 of 10 RCTs comprising 196 stroke patients (116 G-CSF, 80 placebo), mean age 67.1 (SD 12.9), 92% ischaemic, median NIHSS 10 (IQR 5–15), randomised 11 days (interquartile range IQR 4–238) post ictus; data from three commercial trials were not shared. G-CSF did not improve mRS (ordinal regression), odds ratio OR 1.12 (95% confidence interval 0.64 to 1.96, p = 0.62). There were more patients with a serious adverse event in the G-CSF group (29.6% versus 7.5%, p = 0.07) with no significant difference in all-cause mortality (G-CSF 11.2%, placebo 7.6%, p = 0.4). Overall, G-CSF did not improve stroke outcome in this individual patient data meta-analysis.

The impact of stroke on individuals, carers and society is huge and is the third leading cause of death worldwide^1^. Recent progress in acute treatments is encouraging (e.g. mechanical thrombectomy) but they can often only apply to a small proportion of the stroke population. Beyond the acute phase there are very few effective treatments and novel approaches are required.

An ischaemic stroke leads to mobilisation of CD34+ haematopoietic stem cells (HSC), which occurs in bursts over the first 10 days post stroke^2^,^3^; those with higher levels of CD34+ cell mobilisation have a better neurological outcome^2^. Intentional recruitment of CD34+ HSCs from bone marrow to peripheral blood with granulocyte-colony stimulating factor (G-CSF) is a clinical process termed peripheral blood stem cell (PBSC) mobilisation. Although the mechanism is poorly understood, G-CSF alone or with chemotherapy is used routinely in clinical practice to reduce the duration of neutropenia in patients with haematological disease, or for mobilising and harvesting HSCs for subsequent autologous or allogenic infusion. Its use in stroke is under investigation in both animals and humans.

In experimental ischaemic stroke, a number of groups have demonstrated G-CSF to be neuroprotective at various doses^4^, in the presence of thrombolysis^5^, induce functional recovery^6^ and promote angiogenesis and neurogenesis^7^,^8^. G-CSF given early causes a reduction in stroke lesion volume^9^. Consequentially, the use of G-CSF in stroke has progressed into phase II/III clinical trials analysing the effects of G-CSF in hyperacute, acute, subacute and chronic stroke. We have therefore performed an individual patient data meta-analysis on the effects of G-CSF on stroke with the following aims:

(1) To assess the safety of G-CSF administered after ischaemic and haemorrhagic stroke in an individual patient data meta-analysis.

(2) To assess the efficacy of G-CSF treatment after ischaemic and haemorrhagic stroke.

(3) To assess the effect of time of administration on safety and efficacy.

## Results

The initial search highlighted 310 publications; once duplicates, non-stroke studies, experimental studies and review articles were excluded, a total of 10 randomised controlled trials were identified (Table 1)^10^,^11^,^12^,^13^,^14^,^15^,^16^,^17^,^18^,^19^. We received individual patient data from 5 trials^10^,^14^,^15^,^17^,^19^,^20^ and there was sufficient detail in the primary publication of another trial to be included in the analysis^11^. Risk of bias in the included studies has been previously reported in our Cochrane review^21^, which did not include results from one trial included in this analysis^19^.

One study could only be identified as an abstract (so was not included after attempted contact with the author)^12^, and there was no response from the Chief Investigators of three commercial trials (company Axaron/Sygnis) following repeated attempts^13^,^16^,^18^. A recent meta-analysis of Chinese origin describing results in favour of G-CSF includes 4 Chinese publications that did not appear in our systematic searches^22^. Attempts to obtain further detail on these publications from the authors were unsuccessful.

Data comprising 196 stroke patients (116 G-CSF, 80 placebo) revealed a mean age 67.1 (standard deviation, SD 12.9), 92% ischaemic stroke, mean NIHSS 10.3 (SD 5.8), and randomisation at 11 days (interquartile range, IQR, 4–238) post ictus (Table 2). Although the data were more limited, the groups appeared to be reasonably well matched for stroke risk factors including hypertension, diabetes and dyslipidaemia (Table 2).

In univariate and covariate-adjusted analyses (ordinal logistic regression), there was no significant difference between treatment with G-CSF and placebo for end-of-trial mRS: odds ratio (OR) 1.12, 95% confidence interval (CI) 0.64 to 1.96 (p = 0.69), Table 3. There was also no significant difference between groups in NIHSS or Barthel Index (analysed by ANCOVA). A total of 16 (8%) haemorrhagic strokes meant there were too few cases to perform analysis of the effects of G-CSF by stroke pathology. Treatment with G-CSF did not significantly affect outcome (mRS) in different stroke subtypes according to the Oxford Clinical Stroke Project (OCSP) classification^23^, when compared to placebo (data not shown). There was a non-significant trend towards improved Health Utility Status scores in the treatment group (adjusted mean difference 0.088, p = 0.11, Table 3).

**Figure 1:**
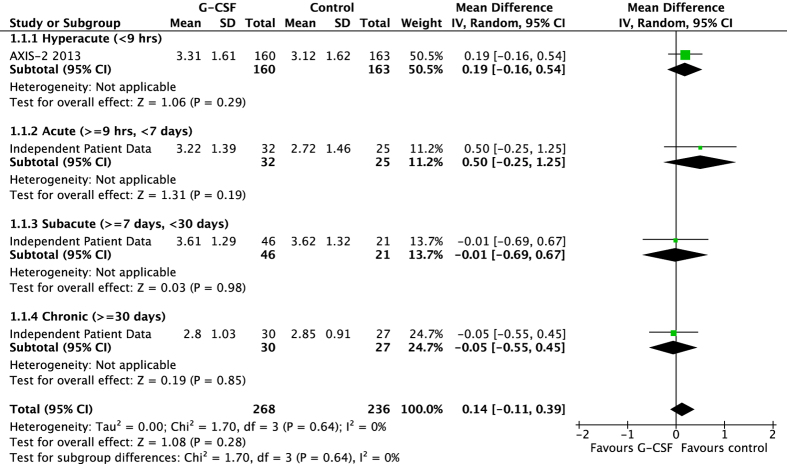
The effect of time of administration on end-of-trial modified Rankin scale according to treatment group; subgroups are divided into hyperacute (<9 hours), acute (9 hours to <7 days) subacute (7 to 30 days) and chronic (>30 days) phases of stroke.

Ordinal analysis indicated a mild but significant influence on outcome in those whom received the treatment/placebo at later time points, OR 1.002, 95% CI 1.0004 to 1.0037 p = 0.015. There was no significant interaction, however, between treatment allocation and time of administration (p = 0.40) within the ordinal model. The effect of time of administration is also represented in Fig. 1 whereby the end-of-trial mRS scores are categorised by time-to-treatment. The data represents mean differences in mRS scores between treatment and placebo groups and is subdivided into hyperacute, acute, subacute and chronic administration times. The figure demonstrates chronic administration trending towards favouring G-CSF, and earlier hyperacute and acute administration trending to favour placebo. Of note, in this summary analysis, we have included data from AXIS-2^18^ despite being unable to obtain individual patient data from the commercial sponsor; we extracted the mean mRS score from each treatment group in this study and calculated the standard deviation from confidence intervals published, thereby providing the data in the hyperacute category.

There were more serious adverse events in the G-CSF group, not reaching significance (p = 0.07, unadjusted analysis); rate of death did not differ between the groups (G-CSF 7% vs. control 4%, p = 0.34, Table 3). There were no reports of new or recurrent haemorrhagic strokes in either group (although one suffered with haemorrhagic transformation of infarction in the control group) and there was no significant difference in the number of vaso-occlusive events, incorporating arterial ischaemia and veno-occlusive disease, by end-of-trial. The distribution of the timing of the vascular events, relative to the first dose of G-CSF, did not differ between groups (log rank test p = 0.51). There was no significant relationship between peak white cell count and vascular events.

## Discussion

G-CSF offers a potential multimodal therapy for both ischaemic and haemorrhagic strokes and this individual patient data meta-analysis has highlighted a number of areas requiring further exploration. Overall, G-CSF had a neutral impact on functional outcome, the modified Rankin score. The time of G-CSF administration significantly influenced functional outcome but no interaction between G-CSF and time was observed, which might be expected if this significant finding was secondary to a G-CSF treatment effect. Patients treated with G-CSF were 1.8 times more likely to suffer from a serious adverse event but this was not statistically significant (p = 0.11 in adjusted analyses).

To determine the optimal time of G-CSF administration, it is best considered in two distinct paradigms: G-CSF enhancing neuroprotection or neurorepair. Two randomised controlled trials have explored the former, AXIS 1 and 2^18^,^24^, administering G-CSF in the hyperacute phase. AXIS-1 reported a small safety study in 44 patients within 12 hours of stroke onset, whilst the follow up trial, AXIS-2, enrolled 328 patients with ischaemic stroke in the MCA territory within 9 hours. There was no difference in efficacy between treatment and placebo groups: G-CSF mean mRS 3.31 (95% CI 3.06–3.56) vs placebo mRS 3.12 (95% CI 2.87–3.37). There were no significant differences in safety or mortality between groups though the absolute number of deaths was higher in the treatment group (22% vs 18%, p = 0.4). These neutral results suggest that assessment of G-CSF in the hyperacute phase of stroke is unlikely to continue. A potential reason for treatment failure could simply be due to giving the drug too late; the preclinical data suggests efficacy if given with 4 hours post onset^9^, whilst mean time to treatment in AXIS-2 was 7 hours. The inability to translate promising pre-clinical treatments to the bedside is also a likely consequence of the experimental models not adequately simulating human stroke. Whilst G-CSF has been shown to be effective in aged rodent models and in models with co-morbidities^25^,^26^,^27^, the problems of age and co-pathologies are often concurrently present in clinical studies, which may be a significant factor in the failure of phase II/III trials. Furthermore, the aging brain is more susceptible to ischaemic damage, demonstrating earlier inflammatory responses to ischaemia^28^ and impaired neurogenesis in the peri-infarct area^29^, which can potentially inhibit neurological recovery.

Chronic administration tests the concept of neurorepair, recovery derived through mobilisation of peripheral blood stem cells (or CD34+ cells). Most pre-clinical data, however, have been developed in models that administer the drug in the hyperacute phase^9^, and the mechanism of recovery is thought largely to be secondary to attenuation of apoptosis in the ischaemic penumbra^7^. However, independent of this, neurogenesis in areas remote to the infarct is seen following treatment with G-CSF^30^. G-CSF also promotes neurogenesis and angiogenesis in peri-ischaemic areas^8^.

Preclinical studies of G-CSF administration in the subacute phase have been performed with additional haematopoietic cytokines. G-CSF in combination with stem cell factor (SCF) given daily 11–20 days post-stroke produced significant improvements in functional and cognitive outcomes when compared to both control and administration in the acute phase (days 1–10)^31^. Similarly, G-CSF and SCF combined induced significant and sustained functional recovery in stroke rats with administration as late as 3.5 months post ictus^32^. More recently, however, experimental studies assessed G-CSF in post-stroke aged rats in combination with bone marrow-derived mononuclear cells^33^ and pre-differentiated mesenchymal cells^34^; 28 days of combined treatment did not enhance recovery in comparison to G-CSF alone in either experiment, further questioning the capability of the aged brain to respond to regenerative therapies.

An area of concern in human clinical studies is the potential risk of G-CSF to induce thrombotic events secondary to an inevitable leucocytosis, and therefore risk of exacerbating recurrent ischaemic stroke or inducing vascular events. Leucocytosis increases with repeated daily dosing and subsequently returns to normal within 5 days of the final dose^10^,^17^. Thrombotic complications would therefore be more likely to occur over the first 5–20 days after randomisation; our data depicts no differences in vascular event rates during this initial time period and no difference overall between groups until end-of-trial follow-up. A second area of safety concern is with increased risk of intracerebral haemorrhage as seen in other trials of colony stimulating factors (erythropoietin)^35^, though there is conflicting preclinical literature as to whether G-CSF attenuates or potentiates risk of haemorrhage when used in conjunction with thrombolysis^36^,^37^. There were no haemorrhagic events in the treatment group of this patient safety set, and none reported in AXIS-2 where two thirds of the cohort was thrombolysed^18^.

Our study has a number of limitations to consider. First, one company that ran three studies failed to respond to repeated requests to share data with the collaboration^16^,^18^,^24^. In particular, absence of the largest dataset (AXIS-2) could have confounded our analyses. We have attempted to overcome this by including their summary data in the subgroup analyses (Fig. 1). One of these study assessing G-CSF in the chronic phase (n = 41) is also absent^16^. There are published concerns about this study: motor function assessments were initially conducted 3 to 7 days after treatment had started (i.e. no pre-treatment values) and participants had almost completely recovered from their initial stroke deficits. Second, our overall sample size is relatively small meaning any findings could be due to chance. Third, the heterogeneity in trial design, such as route and dose of G-CSF and study quality^21^ could have lead to either over- or under- estimates of treatment efficacy and safety.

In summary, G-CSF did not improve stroke outcome in this individual patient data meta-analysis. There are insufficient data on G-CSF administration in the subacute and chronic phases of stroke and further clinical trials should be considered. It seems sensible to adopt an administration time when the acute and potentially toxic inflammatory reaction has started to settle, and treat when the microenvironment favours a remodelling and neuroreparative phase. We suggest a period up to 4 weeks post stroke when most patients are still receiving active stimulus with a rehabilitation programme, perhaps further enhancing neuroregeneration^19^. A trend to an increase in serious adverse events in the G-CSF group highlights the importance of continued safety surveillance in future studies.

## Methods

### Identification of relevant trials

Randomised controlled trials of G-CSF and stroke were sought using electronic searches (Cochrane Library, Medline, Embase, PubMed) up to May 2016, and in a Cochrane Collaboration review of colony stimulating factors and stroke^21^. Key search terms included granulocyte-colony stimulating factor, G-CSF, ischaemic and haemorrhagic stroke, and randomised controlled trial (exploding the search terms). PRISMA guidelines for reporting have been followed.

### Target trials

Randomised controlled trials of G-CSF given in the hyperacute (<9 hours), acute (9 hours to <7 days), subacute (7 to 30 days), and chronic (>30 days) phases of stroke, and involving participants with ischaemic stroke and/or spontaneous intracerebral haemorrhage.

### Data acquisition

Investigators and authors were approached to share individual patient data from their respective trials including: patient demographics-age, sex; risk factors-hypertension, atrial fibrillation, diabetes, ischaemic heart disease, previous stroke; stroke details-date and time, subtype, severity (National Institutes of Health Stroke Scale, NIHSS); trial design-blinding; treatment-start date, length of treatment, treatment received, length of follow up; and outcomes and their date-functional (modified Rankin Scale [mRS], Barthel Index [BI]), impairment (NIHSS), quality of life (Euro-Qol-5D, as health utility status [HUS]), haematology (leucocyte count), vascular events (ischaemic stroke, haemorrhagic stroke, myocardial infarction, systemic embolus, venous thrombo-embolism), and serious adverse events.

### Outcome measures

The number of participants with a serious adverse event, arterial and venous vascular events, and death were key components of safety. Functional outcome was assessed by the mRS at final follow-up. Other outcomes included NIHSS, BI, number of people with an infection and health utility index.

### Data analysis

Following receipt of individual patient data from corresponding chief investigators, the information was tabulated, checked for errors and compared to the primary publication of respective trials. Individual patient data were merged to form a common data set. One trial collected information on stroke severity using the Scandinavian Stroke Scale^10^; these data were converted to the equivalent NIHSS score using a published formula^38^. Univariate and multiple variable analyses were performed, with the latter adjusted for age, sex, severity (NIHSS) and time to treatment. The effects of time-to-treatment were analysed in an ordinal logistic regression model, with mRS set as the outcome variable (ordinal shift analysis), and age, sex, NIHSS, time-to-treatment and treatment-time interaction (trt*time) as the predictor variables. Safety and efficacy were assessed in pre-specified sub groups: stroke subtype and time to administration. Statistical significance was taken at p < 0.05. No data was imputed for missing values; patients who had died were assigned scores of −1 for BI, 6 for mRS, and 43 for NIHSS.

## Additional Information

**How to cite this article**: England, T. J. *et al*. Granulocyte-Colony Stimulating Factor (G-CSF) for stroke: an individual patient data meta-analysis. *Sci. Rep.* **6**, 36567; doi: 10.1038/srep36567 (2016).

**Publisher’s note**: Springer Nature remains neutral with regard to jurisdictional claims in published maps and institutional affiliations.

## Figures and Tables

**Table 1:** Trial design of identified randomised controlled trials of G-CSF and stroke.

Study	Design	Participants	Interventions	Time of administration	Comments on the study
Hyperacute administration					
AXIS 2010*	Double blind RCT, dose escalation	N = 44, ischaemic MCA stroke	G-CSF (Filgrastim), i.v. 30-180 μg/kg or placebo over 3 days	<12 hours	G-CSF appears safe
AXIS-2 2013*	Double blind RCT	N = 328 ischaemic MCA stroke	G-CSF (Filgrastim), i.v. 135 mcg/kg or placebo	<9 hours	G-CSF appears safe. No beneficial effect of G-CSF observed
Acute & subacute administration					
Prasad 2011	Open label RCT	N = 10, ischaemic stroke	G-CSF (Filgrastim) s.c. 10 μg/kg or placebo for 5 days (no placebo)	<7 days	G-CSF appears safe
Shyu 2006	Single blind RCT	N = 10, ischaemic stroke	G-CSF (Filgrastim) s.c. 15 μg/kg or placebo for 5 days	<7 days	G-CSF appears safe. Improvement in NIHSS, ESS and BI at 12 months
STEMS-1 2006	Double blind RCT, dose escalation	N = 36, ischaemic stroke	G-CSF (Filgrastim) s.c. 1-10 μg/kg or placebo for 1 or 5 doses	7 to 30 days	G-CSF mobilises PBSCs post stroke, appears safe
STEMS-2 2010	Double blind RCT	N = 60, ischaemic or haemorrhagic stroke	G-CSF (Filgrastim) s.c. 10 μg/kg or placebo (2:1) for 5 days	3 to 30 days	G-CSF appears safe. PBSCs tracked *in vivo*
STEMTHER 2010	Open label RCT	N = 20, ischaemic stroke	G-CSF (Leukostim) s.c. 10 μg/kg for 5 days (no placebo)	<48 hours	G-CSF appears safe
Zhang 2006*	Double blind RCT	N = 45, ischaemic stroke	G-CSF 2 μg/kg s.c. for 5 days	<7 days	Improved NIHSS by day 20 in the G-CSF group. Abstract only.
Chronic administration					
Floel 2011*	Double blind RCT	N = 41, ischaemic stroke	G-CSF (Filgrastim) s.c. 10 μg/kg or placebo for 10 days	>4months	Feasible and safe administration
STEMS-3	Double blind 2 × 2 factorial RCT	N = 60, ischaemic or haemorrhagic stroke	G-CSF (Filgrastim) s.c. 10 μg/kg or placebo s.c. for 5 days, & PT vs. no PT	3 months to 2 years	G-CSF appears safe in chronic stroke and improves quality of life

^*^Not included in the independent patient data analysis; RCT, randomised controlled trial; MCA, middle cerebral artery; i.v, intravenous; s.c., subcutaneous; NIHSS, National Institutes of Health stroke scale; ESS, European Stroke Scale; BI, Barthel index; PBSC, peripheral blood stem cells; PT physiotherapy.

**Table 2:** Baseline characteristics.

	Placebo	G-CSF	All
	n = 80	n = 116	n = 196
Age	66 (13.1)	67.8 (12.8)	67.1 (12.9)
Male	45 (56.3)	65 (56)	110 (56)
Days from stroke	11.5 [5–286]	10 [4–120]	11 [4–238]
Type			
Ischaemic	73 (91.3)	107 (92.2)	180 (92)
Haemorrhagic	7 (8.8)	9 (7.8)	16 (8)
Baseline NIHSS	9.6 (5.5)	10.7 (6.1)	10.3 (5.8)
	n = 67	n = 99	n = 166
Hypertension	44 (65.7)	64 (64.6)	108 (65)
Diabetes	13 (19.4)	21 (21.2)	34 (20.5)
Dyslipidaemia	30 (44.8)	48 (48.5)	78 (47)
	n = 62	n = 94	n = 156
Atrial Fibrillation	13 (21)	17 (18.1)	30 (19.2)
Previous stroke	12 (19.4)	21 (22.3)	33 (21.2)
Previous TIA	9 (14.5)	14 (14.9)	23 (14.7)
IHD	14 (22.6)	24 (25.5)	38 (24.4)
PVD	2 (2.2)	2 (3.1)	4 (2.6)
OCSP Classification			
LACS	15 (18.8)	19 (16.4)	34 (17.3)
PACS	18 (22.5)	32 (27.6)	50 (25.5)
TACS	26 (32.5)	39 (33.6)	65 (33.2)
POCS	3 (3.8)	4 (3.4)	7 (3.6)

N(%); median [interquartile range]; IHD, ischaemic heart disease; PVD, peripheral vascular.

disease; TIA, transient ischaemic attack; OCSP, Oxford Clinical Stroke Project; LACS, lacuna.

syndrome; PACS, partial anterior circulation stroke; TACS, total anterior circulation stroke.

POCS, posterior circulation stroke.

**Table 3:** The effect of G-CSF compared to placebo on secondary outcome measures.

	Placebo	G-CSF	Unadjusted Odds Ratio or Between-Group Difference (95% CI)	P value	Adjusted Odds Ratio or Between-Group Difference (95% CI)*	P value
	n = 79	n = 116				
N° with SAE†	14 (7.5)	34 (29.6)	1.93 (0.95 to 3.89)	0.07	1.82 (0.89–3.75)	0.10
Death end of trial†	3 (3.8)	8 (7.0)	1.88 (0.48 to 7.3)	0.36	1.49 (0.36–6.16)	0.59
Vascular occlusive events†	6 (7.6)	12 (11.2)	1.42 (0.51 to 3.96)	0.52	1.21 (0.43–3.45)	0.72
	n = 73	n = 109				
End of trial mRS‡	3.03 (1.3)	3.26 (1.3)	1.3 (0.8 to 2.2)	0.24	1.12 (0.64 to 1.96)	0.62
	n = 72	n = 114				
End of trial NIHSS§	7.7 (8.5)	9.2 (10.7)	1.6 (−1.5 to 4.5)	0.30	0.7 (−1.9 to 3.2)	0.62
	n = 75	n = 114				
End of trial BI§	67.1 (29.6)	63.3 (34.4)	−3.8 (−13.4 to 5.7)	0.43	−0.5 (−7.6 to 6.7)	0.90
	n = 56	n = 83				
Health Utility Index§	0.392 (0.364)	0.463 (0.338)	0.071 (−0.048 to 0.19)	0.24	0.088 (−0.019 to 0.195)	0.11
	n = 80	n = 116				
Peak WCC	7.46 (3.46)	31.34 (17.54)	23.88 (19.96–27.8)	<0.0001	23.83 (19.87–27.8)	<0.0001

Data shown are number (%) for categorical events, mean (standard deviation) for mRS, NIHSS and BI.

^*^Adjusted for age, sex, baseline NIHSS and time-to-treatment. Analysed by

^†^Logistic regression

^‡^ordinal logistic regression and

^§^ANCOVA. mRS, modified Rankin scale; NIHSS, National Institutes of Health Stroke Scale; BI, Barthel index; SAE, serious adverse event; OR odds ratio; CI, confidence interval; WCC, white cell count.
